# Navigating the Tensions of Supervision: Medical Educators’ Strategies for Supporting Students During End-of-Life Care

**DOI:** 10.5334/pme.1623

**Published:** 2025-11-05

**Authors:** Diego Lima Ribeiro, Daniele Pompei Sacardo, Grazyna Drzazga, Marco Antonio de Carvalho-Filho

**Affiliations:** 1 University Medical Center Groningen, University of Groningen, The Netherlands; 2 Public Health Department, State University of Campinas, Brazil; 3 Department Public Health, University of Campinas, Campinas, Brazil; 4 Lifelong Learning, Education, and Assessment Research Network (LEARN), University of Groningen, Groningen, The Netherlands; 5 Wenckebach Institute (WIOO) –Lifelong Learning, Education, and Assessment Research Network (LEARN), University Medical Center Groningen, Groningen, The Netherlands

## Abstract

**Introduction:**

Medical educators are central to guiding students through the emotional challenges of moral dilemmas in end-of-life (EoL) decisions. However, the literature often overlooks how medical educators support students during these moments. This study explored strategies medical educators employed and challenges they encountered in supporting students facing moral dilemmas in EoL care.

**Method:**

This qualitative study analyzed interviews with fifteen medical educators from diverse clinical settings, focusing on how they support students during EoL decisions to withhold or withdraw life-sustaining therapy.

**Results:**

Medical educators initially offered equal support to all students but progressively adapted their strategies based on perceived student engagement. These strategies were not pre-planned or fully conscious, but emerged in real time as educators navigated the emotional and cognitive demands of EoL decisions. Medical educators increased personalized support to students perceived as “highly engaged”, while gradually reducing support for those perceived as “less engaged”. They struggled to balance their role as comprehensive supporters with time constraints, administrative duties, and a hidden curriculum prioritizing clinical skills over emotional connections and patient-centered care.

**Discussion:**

Building on our previous investigation into medical students’ experiences of moral dilemmas in EoL care, this study shifts the lens to medical educators. It reveals a critical oversight: students feeling emotionally overwhelmed may be misinterpreted as disengagement. This misalignment can hinder educators’ support strategies, potentially reducing support when most needed. The study advocates for an ‘Educational Alliance’ grounded in trust and mutual respect, and calls for structural reforms to sustain educators’ capacity to provide consistent, emotionally attuned support.

## Introduction

In recent decades, there has been a significant increase in the autonomy of seriously ill patients regarding decisions to withdraw or withhold life-sustaining treatments [1,2,3]. These decisions often trigger moral dilemmas and evoke intense emotional reactions among all healthcare professionals [4,5], particularly impacting medical students [6,7]. As medical educators stand on the front line of end-of-life (EoL) care, they are tasked with navigating these dilemmas themselves as well as preparing future physicians to manage such situations adeptly [8,9,10]. Despite their crucial role, medical students report feeling unsupported by their supervising physicians in coping with the emotional weight of EoL decision-making [11]. In a previous study, we explored students’ experiences with EoL dilemmas and found that many felt emotionally overwhelmed and silenced by a hidden curriculum that discouraged emotional expression [12]. Students felt alone while struggling to make a choice between detaching from patients or connecting with them [12,13]. Building on these findings, the present study shifts the focus to medical educators [13,14]. We sought to understand how educators perceive and enact their supportive role — and what tensions, dilemmas, and constraints they face in doing so. By examining their strategies and contextual constraints, we aimed to explore how educators’ perspectives and actions align (or potentially misalign) with students’ emotional needs. We believe that by better understanding educators’ support strategies, we can inform future interventions that more effectively address students’ needs.

The realm of EoL care presents medical students with real-world complexities, such as sharing the diagnosis of incurable diseases with patients and making critical care decisions, including whether to withhold or withdraw life-sustaining treatment [11,15]. Withholding life-sustaining therapy involves not starting new treatments when death is inevitable [16,17], e.g., refraining from initiating hemodialysis for a patient with renal failure in the context of inevitable death. Withdrawing life-sustaining therapy entails stopping ongoing interventions [6,17], such as discontinuing vasoactive drugs from a patient who is deteriorating progressively despite optimal clinical care. Engaging with such complex decisions surrounding life-sustaining treatment, combined with the emotional weight accompanying them, underscores the importance of both educational guidance and emotional support from medical educators [18].

Medical educators play a pivotal role in bridging the gap between theoretical knowledge and practical experience, especially in the emotionally charged context of EoL care [10]. Their role extends beyond imparting knowledge; it includes helping students cope with their emotional reactions and navigating the ethical tensions that arise during these critical decisions [19,20]. In this study, we use the term *support* to refer to both educational guidance (e.g., clinical discussions, didactic input) and emotional scaffolding (e.g., attentive listening, emotion regulation), which means that, in clinical settings, supporting students includes modeling compassionate communication, facilitating moral reflection, and creating psychologically safe spaces for dialogue [21,22]. Understanding how educators perceive and implement these supportive strategies may offer insights into how they respond to students’ emotional and moral struggles — particularly when those struggles arise from complex moral dilemmas in the context of EoL care.

In a prior study, we elaborated on the complexity of the moral dilemmas faced by medical students, which involve ethical considerations, cultural values, and personal beliefs [23]. We define a moral dilemma as a situation where students navigate conflicting values when making a clinical decision, where each option leads to actions that seem incompatible, with none appearing sufficient [23,24]. These dilemmas often leave students feeling overwhelmed, not only triggering emotional reactions like frustration, anger, and anxiety but also instilling a pervasive sense of uncertainty [19,23]. Ideally, medical educators should prepare students to engage with such difficult decision-making processes and cope with the emotional turmoil accompanying them [25]. This dual responsibility presents a unique challenge in medical education [26,27], emphasizing the need for a nuanced understanding of how educators support their students.

Despite the critical need to support medical students navigating moral dilemmas in EoL care [7,23,24], there remains a substantial gap in research concerning how medical educators experience and enact this responsibility. Existing literature tends to overlook the emotional labor, ethical tensions, and contextual constraints educators face when supporting students in these challenging situations. To address this gap, we investigated the following question: How do educators support medical students when navigating moral dilemmas during end-of-life care? By illuminating educators’ experiences, challenges, and adaptative strategies, this study aims to inform systemic changes in faculty development that recognize and address the ethical, emotional, and educational complexities of supervising students in EoL care.

## Methods

### Design

We conducted a cross-sectional qualitative study rooted in a constructivist paradigm, which embraces the diversity of interpretations of reality [28]. We applied thematic analysis [29] to a data corpus consisting of one-on-one interviews with medical educators teaching EoL care to undergraduate medical students. This study received ethical approval from the research ethics committee of the State University of Campinas (CAAE: 55505121.2.0000.5404).

### Context

We carried out the study at a Brazilian medical school, where the medical course spans six years, with the last three years primarily devoted to clinical practice in clinical wards, operating rooms, outpatient clinics, emergency departments, and intensive care units. During this phase, students have prominent roles in patient care, often assuming a protagonist-like role. This early and intensive clinical involvement prepares students for autonomous practice at primary care units and emergency departments immediately after the undergraduate course, in accordance with Brazilian regulations. This pressure to produce autonomous doctors at the end of the undergraduate course compels medical educators to become flexible and adapt their supervision to the diverse students’ needs. While there is a standard amount of time to be dedicated to each group of students in clinical rotations, if students show commitment and engagement with the learning activities, medical educators often make extra time for these students and unlock greater opportunities for hands-on practice, case discussions, and direct patient care. This additional time could range from extra minutes or hours added to the end of regular rounds to additional sessions specifically designed for certain motivated student groups. This flexibility reflects a common and pragmatic approach in Brazilian clinical education. At the same time, it introduces a dynamic in which students’ access to learning opportunities may vary — a context that could offer valuable insights into how educators informally support students during critical decisions in EoL care.

### Participants

We adopted a purposeful sampling strategy to ensure a diverse representation of clinical perspectives related to end-of-life (EoL) care. The first author (DLR), who has longstanding clinical and teaching experience in this same institutional context, identified and invited educators from various specialties, genders, ages, and academic backgrounds who had significant experience supervising medical students in EoL care. Our aim was to capture a broad range of views on how educators support students in emotionally and ethically complex situations. Educators were selected from different clinical settings where EoL care frequently takes place in Brazilian university hospitals, such as internal medicine, oncology, emergency medicine, intensive care, and neonatology. The variation in clinical contexts was intentional, as we anticipated that time constraints and different patient needs in each setting might shape the way educators support students. In a few cases, educators were also recommended by colleagues based on their teaching involvement in EoL care, and we included them when they met our inclusion criteria of having at least five years of experience (average = 16) supervising students in such settings. We included individuals across different clinical units, professional trajectories, and academic ranks to ensure a rich and varied dataset (see Table 1). All participants were personally contacted by the first author and provided with detailed information about the study. Written informed consent was obtained from each educator before the interview, in accordance with institutional ethical guidelines.

**Table 1:** Professional profile of EoL care educators at State University of Campinas, Brazil, 2023.


	AGE	GENDER	PROFESSION	SPECIALTY	EDUCATION	YEARS OF EXPERIENCE

**Iw_EoL1**	54	female	Physician	Intensive care/Palliative care	PhD	23

**Iw_EoL2**	40	female	Nurse	Palliative care	PhD	16

**Iw_EoL3**	40	male	Physician	Intensive care	Msc	12

**Iw_EoL4**	32	male	Physician	Emergency physician	–	5

**Iw_EoL5**	42	male	Physician	Pulmonologist	Msc	18

**Iw_EoL6**	39	male	Physician	Internal Medicine	–	14

**Iw_EoL7**	44	male	Physician	Pulmonologist	PhD	18

**Iw_EoL8**	33	female	Physician	Emergency physician	–	5

**Iw_EoL9**	42	female	Physician	Oncologist	Msc	15

**Iw_EoL10**	46	male	Physician	Intensive care	PhD	20

**Iw_EoL11**	60	female	Physician	Neonatologist/Palliative Care	Msc	32

**Iw_EoL12**	45	male	Physician	Neurologist/Intensive care	PhD	16

**Iw_EoL13**	39	female	Physician	Internal Medicine	–	10

**Iw_EoL14**	61	male	Dentist	Oral and maxillofacial surgeon/Bioethics	PhD	32

**Iw_EoL15**	47	female	Physician	Neonatologist/Palliative Care	–	17


### Data collection and sample size

This study serves as a follow-up to our previous research focused on understanding the moral dilemmas faced by medical students during EoL care [12]. In the preceding study, students created Rich Pictures of EoL experiences marked by moral dilemmas. These rich pictures were complemented by interviews that provided a foundation for our understanding and served as a pivotal reference for the development of this current study. Our interviews with medical educators, conducted from June to December 2023, started during the final stages of students’ interviews from the previous study. This timing was strategic, allowing insights gathered from students to inform and refine our interview protocols for educators directly.

For the current study, DLR, who was responsible for data collection, shared the definitions of moral dilemmas and the concepts of withholding and withdrawing life-sustaining therapies with participants (see Supplementary material– Interview Guide). Presenting these definitions was essential to ensure a shared understanding of the key concepts central to our research questions. The initial interview guide was developed based on the student data and focused on how educators perceived students’ experiences. However, the guide evolved iteratively [29] throughout the study as new insights emerged from early educator interviews. For example, as themes such as differentiated student engagement and the role of institutional constraints emerged, the interview guide was adapted to explore these areas in greater depth. Later additions to the guide included more targeted questions, such as “Can you describe a specific strategy or approach you use to support students in morally complex end-of-life situations?” and “Have you ever experienced a moment when you felt unable to offer the support a student needed?” These additions were designed to directly align with the study’s aim of exploring educators’ support strategies and were informed by both early educator interviews and ongoing insights from our concurrent student research. The first five interviews followed the initial version of the guide, while the remaining ten incorporated these emergent themes through revised questions. This flexibility allowed us to probe educator strategies in context, without imposing rigid or pre-formulated assumptions. A revised version of the interview guide, with added and removed questions marked, is provided in the Supplementary File to enhance transparency (Supplementary material– Interview Guide).

Our sampling strategy drew upon the concept of information power, which considers the adequacy of the sample in relation to the study’s aim, the specificity of the sample, the quality of dialogue, and the depth of analysis [30,31]. We focused on a clearly defined aim — understanding how educators support students during moral dilemmas in EoL care — and interviewed a relatively specific and experienced group of medical educators involved in supervising students in these settings. The interviews were in-depth and reflective, and the analysis followed a systematic and iterative approach.

As the study progressed, the research team regularly reviewed the interviews and evolving themes. After 15 interviews, we found that the material provided sufficient diversity and depth to develop meaningful interpretations of educator support strategies across different clinical settings and institutional roles [30,31].

### Data analysis

The research team conducted a thematic analysis in six defined steps, following Braun and Clarke’s approach [32]. First, DLR and DS independently scrutinized the first six transcripts, ensuring a thorough initial understanding. Second, they crafted an initial coding framework directly influenced by the research question— *“How do educators support medical students when navigating moral dilemmas during end-of-life care?”* Through weekly meetings, they rigorously evaluated and refined these codes, allowing for the dynamic addition, modification, or removal of codes as necessary. MACF’s involvement in these meetings deepened the team’s comprehension of the codes and highlighted data gaps needing further investigation. MACF, for instance, facilitated discussions on what constitutes moral judgments and behavior-based assessments, among other insights that helped refine the coding process. In the third step, the authors collaboratively identified themes through an iterative process, meticulously comparing and contrasting interpretations to achieve consensus on the initial themes. This continuous comparison of new data and interpretations against previous assumptions and understandings not only refined their thematic framework but also enhanced subsequent interviews, leading to the next step. Fourth, the research team reflected on the analysis of the first six interviews to refine subsequent interview topics [33]. This reflection revealed that educators categorize students into groups, tailoring support strategies accordingly. In parallel, the team noticed that many educators also shared spontaneous reflections on their own emotional, cognitive, and institutional challenges in enacting these support strategies. Although these struggles were not the initial focus of the interview guide, they emerged consistently across interviews and were incorporated into the ongoing analysis. Subsequent interviews by DLR explored both the classification criteria and the broader context shaping educators’ support efforts. DLR maintained an open dialogue, practiced active listening, and skillfully navigated the conversation to deepen the analysis based on previous responses. GD joined the group in this step, reading the first four translated interviews. The team addressed any translation uncertainties in regular meetings, further elaborating on the themes, providing insights, and constructing new meanings that guided the new interviews. In the fifth step, the research team engaged in profound discussions to further dissect the elements, contexts, mechanisms, and interrelations characterizing educators’ support strategies and their influencing factors. This collaborative effort enabled them to weave a coherent and insightful narrative that elucidated the themes and their interconnections. Finally, the sixth step involved collective efforts in manuscript writing. DLR meticulously documented the coding process, theme development, and the creation of mind maps in a logbook. Additionally, the research team’s meetings (recorded by DLR) served as critical reflective forums, ensuring the rigor of data analysis.

### Research group and reflexivity

Our research team brought together individuals with diverse yet complementary expertise, essential for the nuanced analysis of our data. DLR, serving as an internal medicine specialist with clinical and teaching experience in EoL care, has worked across various units—emergency department, internal medicine ward, and intensive care unit—at the institution where data was collected. His firsthand experience with EoL decision-making and the dying process offered an insider’s view that enriched our study with deep, personal insights. DS, a psychologist and bioethics professor at the same institution with a specialization in identity and moral development, provided critical psychological and ethical reflections within our analysis. GD, a cognitive linguist with a medical education and faculty development background, contributed with a distinctive blend of professional and cultural perspectives. As an outsider to the clinical realm, GD’s expertise in qualitative research challenged us to question established assumptions and reframe familiar concepts, enhancing their relevance and accessibility. Finally, MACF, a professor of health profession education research with previous experience with supervising students and residents in EoL care, has a research focus on transition to practice, which encompasses our research question. His extensive experience with terminally ill patients in under-resourced clinical settings provided him with a profound understanding of the practical, emotional and clinical challenges faced in such environments. The integration of clinical and educational perspectives enabled MACF to contribute significantly to our study by bridging the gap between theoretical knowledge and practical application in real-world scenarios.

## Results

While our initial focus was on how educators support students, our findings revealed that these support strategies are embedded within a broader process. Medical educators first acknowledge that EoL decision-making is difficult for students and offer initial support to all of them. They then make sense of students’ behaviors and emotional responses—often through informal classifications of engagement—before deciding how to tailor their support. However, this is not a deliberate strategy, but rather a response embedded within and influenced by the emotional and cognitive demands of the clinical environment. As our data analysis progressed, it became clear that supporting students is inseparable from educators’ own struggles: navigating uncertainty, managing emotional labor, and balancing ideals about what good supervision entails with institutional constraints. The sections that follow reflect this layered reality—one in which understanding, *“judging”*, and supporting students are deeply interconnected.

To understand how support unfolds, we begin by exploring how educators perceive the challenges students face in EoL care and how these perceptions shape their initial approach. Medical educators highlighted the significant challenges students face in EoL care, underscoring the complexity and emotional weight of clinical decisions in this context. In parallel, they identified significant gaps in students’ theoretical knowledge about EoL, noting that students’ scant exposure to terminal patients during their undergraduate studies resulted in a lack of firsthand experience with patient deaths. This gap in knowledge and experience often left students feeling unprepared, insecure, shocked, anxious, and sad.

**Iw_EoL5:** *“The surprise and perplexity stem from it being something very new to them; that is, I don’t see them experiencing during their undergraduate studies a situation of therapeutic limitation. The learning during the pre-clinical education does not have much room to talk about end-of-life care […]. There’s also the aspect of understanding the prognosis of diseases, which nobody talks about […] So, a fundamental part, which involves talking about prognosis, end of life, medical decision-making, ends up having very little space during the undergraduate course.They are not prepared beforehand.”*

When these inexperienced and unprepared students have to navigate the moral dilemmas common to EoL care, the choice between extending a patient’s life and prioritizing comfort measures aimed at alleviating suffering becomes especially challenging. The difficulty of making such choices stems from their inherent uncertainty, which poses a hard challenge for inexperienced students in determining the best course of action. Medical educators attribute this uncertainty to three key factors. Firstly, although terminal illnesses inevitably lead to death, predicting the timing or contributing factors remains uncertain. Secondly, doubts arise when considering whether extending life under a significantly reduced quality of life justifies the interventions required. Finally, personal values play a significant role in determining what is deemed the ‘right’ decision. For instance, while some may perceive a good quality of life as possible even when bedridden, others may view such a state as lacking dignity. The complexity of making choices amid these uncertainties hinders students’ ability to discern the best course of action, generating emotional distress. In this context of clinical uncertainty, moral dilemmas, and emotional burdens, educators are tasked with identifying and implementing effective strategies to support medical students.

**Iw_EoL8:** “*My impression is that students still have a strong sense of right and wrong. The medical approach is taught as merely treating diseases. When it comes to ethical decisions, they are often searching for which decision are right and which are wrong […]Many of them lack the maturity to understand that in many aspects of life, there isn’t a clear right or wrong.”*

In the following sections, we will explore the nuanced process through which educators engage with students when supervising EoL care. First, we will elaborate on how educators classify students by their level of engagement. Second, we will describe how this classification influences the supportive strategies offered to students. Third, we will reflect on how educators balance the emotional labor and cognitive load while devising their supportive strategies. Finally, we will delve into the dichotomy between the idealized concept of supervision by supervisors and its actual implementation, shedding light on the complexity of supervising students during EoL care (see Supplementary Material – Thematic Map of Medical Educators’ Support Strategies in End-of-Life Care).

### Classifying students by engagement level

The classification of medical students into “highly engaged” and “less engaged” categories emerges as one of the most common themes in the educators’ interactions. This classification arises from the observed behaviors, such as participation in case discussions and clinical rounds.

**Iw_EoL3:** *“It depends on the level of engagement the student has towards the patient and the clinical rotation. Some students are more passive, simply listening to what you’re saying and trying not to get involved. There are students who are more engaged […] asking questions and even inquiring—not so much questioning your conduct, but seeking to understand the basis of your decision-making.”*

During the interviews, medical educators often described highly engaged students with terms such as “proactive”, “involved”, and “participative”. They believe that highly engaged students exhibit a keen focus, posing specific and contextualized questions, for instance, asking about medication dosages to alleviate suffering. In clinical settings, such students strategically position themselves to ensure close proximity to colleagues, educators, or patients, facilitating attentive and active listening. Whenever possible, these students dedicate additional time after the clinical rounds are over to extend the conversation with supervisors and peers, diving into meaningful clinical, psychological, and social dimensions of patient care. Highly engaged students also join conversations with patients and their families and pose philosophical questions to educators about death, dying, and the meaning of the medical profession. Educators commend this level of engagement as a clear indication of students’ commitment to their own professional development and patient care.

**Iw_EoL12:** *“So, there’s a non-verbal language, right? They (highly engaged students) look at you, they will look at you and stay focused on what you’re saying. […]the moment you switch to talking about end-of-life care, […] “José”, so many years old, was quite frail when he was admitted. So, what do we plan for him? How far do we go? How are we going to do this? Then, I see that the student pays attention.The gaze is usually eye to eye, stopping what they’re doing to pay attention to this reflection.”*

In contrast, medical educators refer to less engaged students with descriptors such as “passive”, “not involved”, “distanced”, and “disengaged”. These individuals typically remain silent throughout case discussions, adopting the role of observers. Specifically, during clinical rounds, students classified as less engaged keep a notable physical distance, avoiding interaction with patients and peers. They also leave immediately after practical activities conclude, a behavior interpreted by medical educators as a lack of commitment and indifference toward patient care. Some medical educators even use the word “cynical” to describe this perceived detachment.

**Iw_EoL13:** *“There are students who you really notice that they don’t want to get involved. They just don’t engage, period. And that’s it, right? They don’t want to get involved because they don’t want to come into contact with those feelings (sadness, frustration, anguish).”*

**Researcher:** *“And how do you notice that they don’t want to get involved?”*

**Iw_EoL13:** *“It’s mostly through non-verbal language. They keep their distance. In a sense of true indifference, they just agree, don’t challenge, just follow along, you know? […] They agree just to let the situation proceed, they don’t contest, they don’t ask questions.”*

### Supporting students: individualizing trajectories vs. providing the basics

At the beginning of the clinical rotation and before classifying students into “highly engaged” and “less engaged”, educators offer identical teaching and support strategies to all students. However, as they evaluate student engagement, their support becomes tailored: students perceived as highly engaged receive more personalized attention, while support for those perceived as less engaged decreases. We will elaborate further on this differentiated approach to support, contingent on student engagement, in the sections that follow.

#### Initial approach

Acknowledging the significant gaps in medical students’ knowledge, medical educators initially introduce complementary lectures and small group activities focused on the complexities of EoL care. These sessions cover essential topics such as symptom management, communication skills, and the clinical prognosis of prevalent chronic illnesses.

**Iw_EoL1:** *“I believe that students end up dealing with the anxieties related to end-of-life care on their own. I have this feeling, and I think it’s not a good thing. I’ve seen that there’s a lot of distress due to a lack of knowledge; the student doesn’t understand what an end of life without feeding is, saying ‘the patient is going to die of hunger.’ […] To explain, ‘look, at the end of life, the person won’t feel hunger, it’s part of the nature of life,’ to show them first from a technical perspective to see if this can alleviate some of the emotional conflicts.”*

At this stage, educators provide all students with opportunities to engage in direct patient care while also fostering a supportive environment to address the challenge of the context of the EoL decisions. Acknowledging the moral dilemmas students face during EoL decisions, educators nurture a welcoming atmosphere, promote an open dialogue, and offer emotional support. Notably, all participants reported integrating additional debriefing sessions, especially after emotionally charged experiences like a patient’s death. Medical educators strive to create a safe space for students to reflect on their experiences, covering critical topics, including the process of dying, EoL care practices, and delivering bad news. Some educators even adjust their own schedules to allocate extra time for further support. While this initial openness and commitment are available to all students, medical educators also assess student engagement during this phase and adjust their support accordingly.

#### Tailored support for students perceived as highly engaged

Medical educators reported offering personalized support to students classified as highly engaged, emphasizing the transition from group settings to individualized attention. This personalized approach is evident in the creation of safe and confidential spaces for one-on-one sessions, as well as in fostering a welcoming atmosphere through gestures like sharing food, such as chocolates, or inviting students for a coffee. Such acts of care, particularly the tradition of sharing food, aim to cultivate an environment conducive to open the dialogue and create rapport.

**Iw_EoL2:** “*We always make ourselves available for a conversation. Once, a student started crying, and I didn’t know what to say —so I just gave a strong hug and chocolates. We make it clear we’re always there for them; for whenever they face a difficulty or have a question about anything, they should feel free to reach out to us.”*

In this context, medical educators invest in fostering solid, authentic, and trustworthy relationships with their students. For instance, one medical educator recounted moving a discussion with a highly engaged student to a bench in a small garden at the hospital’s entrance. This change of scenery facilitated a deep, meaningful conversation, addressing the student’s immediate concerns and emotional turmoil after participating in EoL care. By doing so, the educator created a safe and confidential space and addressed the specific needs of the student, thereby fostering a trusting relationship and supportive environment. In general, active listening to students’ personal and professional challenges was vital to offer tailored guidance and respect the unique journey of each student. Medical educators expressed such an immense pride in these highly engaged students that, during the interviews, when describing such students, they changed their tone of voice, often speaking louder and smiling.

**Iw_EoL9:** *“I take them out of the hospital and lead them to sit on the benches in the garden in front of the third-floor entrance. […] I’ve even offered to talk to the students over the phone, outside of working hours, but I prefer to do this in person and away from the hospital. I say, ‘Let’s go over there, near a nice, tree-filled place.’ And even though they’re all students, each one has their own individual way. […] Every time, I learn a lot from each of them. I discover the human pain of each one, the way that pain affects each one. Because the sadness they feel has touched each of them in a unique way. Some are touched because they have lived through it […] ; some because they fear it happening to someone they love; some fear being the person who will have to make the decision.”*

#### Basic support for students perceived as less engaged

From the outset, educators strive to motivate those who appear disengaged, inviting them to contribute actively to rounds and discussions; however, when these strategies consistently fail to elicit the expected level of engagement, educators perceive this resistance as a reluctance to embrace the learning opportunities provided. Such observations prompt educators to question these students’ underlying motivation and receptivity to learning. Repeated unsuccessful attempts to engage these students lead to a gradual shift in the medical educators’ approach. They reluctantly begin to reduce direct support for students who are perceived as less interested in learning about EoL care and patient care. This diminished support reflects the transition to maintaining a more superficial relationship. This emotional detachment stems from the disappointment and sense of betrayal triggered by the feelings of frustration originating from the perceived lack of reciprocity, i.e., the perception that students are not investing in the relationship as much as medical educators. This frustration can be so intense that, during the interviews, when talking about the interactions with students perceived as less engaged, participants filled the conversation with pauses, silences, and halting sentences, where the term “difficult” frequently surfaced.

**Iw_EoL11:** *“Sometimes, for instance, I’m talking to someone, and I notice they are, like, crossing their arms, right? Then, suddenly, I stop to think and say, wow, this behavior is typical of someone [laughter, satirical gesture of strangulation]… you’re holding yourself back not to… [laughter, satirical gesture of strangulation] not to jump on the student’s throat [laughter]. You have to be open to this type of attitude from the student [returning to a serious tone]. But, you know, that person who is physically distant from the process, right?[…] Or they look at you with that expression like… ‘I really wish I weren’t here.’ […] I can’t tell you, and that’s my questioning: Is ethics teachable? Because I don’t know if I can make that person who is uninterested, interested. I think it’s complicated to be in the healthcare field and not be concerned about the ethical issues of what you’re going to do, right? I think that’s a serious problem.”*

### Juggling complexities: cognitive load, emotional labor, and student support

Building upon the profound emotional and supportive dynamics between educators and students discussed earlier, medical educators encounter an escalating cognitive burden as they navigate the complexities of EoL decision-making. Initially, they confront uncertainties in decision-making, such as the choice between life-sustaining interventions and palliative care approaches. Despite these difficulties, their accumulated expertise provides them with confidence in navigating those decisions with patients and families. However, the complexity of these decisions is just the tip of the iceberg. A primary challenge for educators lies in transparently sharing these uncertainties with their students, aiming to transform EoL situations into profound learning experiences. This challenge stems from the way students often interpret these inherent uncertainties—as a reflection of clinical incompetence. In a culture that praises certainty, sharing doubts and navigating ambiguous situations is perceived as a sign of failure. In this context, medical educators often perceive a judgmental gaze from their students, further elevating their emotional and cognitive burden to daunting levels. This perceived scrutiny amplifies the challenges medical educators face in navigating EoL decision-making.

**Iw_EoL10:** “*Despite our best efforts—constantly seeking evidence, engaging in discussions about ethics, and studying—people (students) still question our legitimacy.”*

Medical educators navigate a complex emotional landscape in EoL care, where their own emotional experiences intersect with those of patients, families, and the medical team. This emotional spectrum encompasses deep sadness over patient loss, empathy toward family suffering, and witnessing the medical team’s distress. Such circumstances demand substantial emotional labor from educators, who also bear the responsibility of guiding medical students. They mentor these future professionals in managing their emotions while upholding compassionate, patient-centered care. The convergence of these emotional challenges not only escalates the emotional labor for medical educators but also influences the support they offer students. Moreover, medical educators often feel as if they are contributing to the suffering of medical students by exposing them to EoL situations, which trigger feelings of guilt.

**Iw_EoL7:** “*The student has to deal with feelings that could be, for instance, anger, fear, or even guilt, for having felt something, thought something, or been a part of that end-of-life situation. And when we’re the ones triggering these emotions in the students, we have to deal with our own anger or fear of having caused harm. We have to grapple with the guilt of having brought significant emotional distress to the student.”*

### Navigating the tension between idealized support and practical challenges

Medical educators navigate a profound tension between their ideal role as supportive mentors and the harsh realities of their professional environment. They aspire to mentor medical students through the cognitive and emotional challenges of EoL care, embodying the role model they wish to be. However, the reality they face —saturated with lectures, hospital rounds, and administrative tasks—imposes significant barriers to providing the level of personalized support they envision. During the interviews, medical educators highlighted how these competing demands severely limit their capacity for direct, one-on-one engagement with students.

**Iw_EoL10:** “*This was a reflection I’ve had because I think I’ve been secluded in my office in these past years. As I became a member of the faculty, I took on this belief that I needed to focus on research and that I need to fulfill all commitments. And so, I end up spending a lot of time alone in my office, prepping lectures […] and I end up spending very little time in practice with the students. This is something I’ve been thinking a lot about because an educator who isn’t involved in the day-to-day practice doesn’t hold any credibility. So, we need to be present, right? I’m even considering having an honest talk with the faculty department to say that I can’t juggle everything at once; it’s just not fair.”*

Additionally, medical educators shed light on the impact of a hidden curriculum within clinical settings – a set of unofficial and unintended lessons that profoundly shape students’ perceptions and approach to EoL care. This hidden curriculum often emphasizes the acquisition of technical clinical competencies at the expense of focusing on developing emotional regulation strategies and embracing patient-centered care. Medical educators voiced concerns about this skew, feeling it hampers their goal of supporting students through the emotional dimensions of EoL care. They feel unprepared and attribute their lack of readiness to both the hidden curriculum’s focus on technical over emotional aspects and a perceived absence of institutional support in providing resources or training for medical educators to guide and mentor students effectively.

**Iw_EoL14:** “*Pedagogical processes in medicine are heavily weighted toward technical disciplines, leaving little room for the humanistic ones. […] If the supervising doctor doesn’t focus on discussing patient-centered care, the student won’t value it. The same goes for rotations in the ICU; if the intensivist doesn’t emphasize the patient’s values, the student won’t see its importance. This is because ethical decision-making, which delves into morality, is complex. It’s complex because, unlike technical interventions where you might discuss medication dosages or perform a procedure, moral issues require you to weigh why something should be done in a certain way or if there’s an alternative. This kind of deliberation isn’t something the university prepares you for. […] The value we end up teaching is the ‘means’—the procedure, the intervention—but not the ‘end’, which is the patient and reflecting on whether or not a certain procedure should be done. No one teaches us that.”*

This tension between the educators’ idealized support and their lived reality frequently culminates in feelings of guilt and frustration. They wrestle with a sense of inadequacy, stemming from their inability to fully meet the emotional and educational needs of their students. This emotional toll is not merely a result of external pressures but also mirrors internal conflicts within their professional identity—a constant struggle to align their high aspirations with the constraints imposed by their work environment. These feelings of guilt and frustration are intensified by the educators’ acute awareness of the critical importance of their role in shaping students’ competencies in EoL care.

**Iw_EoL7:** “*I keep thinking, where are we failing? And I believe this is where we’re falling short. We tell students that bonds are important (between students, patients, educators and team), but we demand that they arrive early, see the patients (quickly), and then rush off to class… The student’s entire journey is like this […] Then, we tell the student that it’s important to build strong relationships. How can we say that connections are important, do you see? […]It’s utterly contradictory, don’t you agree? … So, how are they (and us) supposed to build relationships?”*

## Discussion

Our research began by exploring how medical educators support students during challenging EoL care decision-making. As the study progressed, however, educators’ support strategies were entangled with their own moral and emotional struggles, perceptions of student engagement, and the institutional conditions in which support takes place. A key insight is that support is filtered through a *tacit lens* of engagement: Medical educators tend to classify students as “highly engaged” or “less engaged”, based on observed behaviors. While this classification is not a deliberate strategy of support, it significantly influences how support is provided. Students perceived as highly engaged benefit from individualized support, while those perceived as less engaged receive less support. However, this binary classification overlooks a crucial aspect: students’ apparent disengagement could stem from the significant emotional and cognitive demands of confronting death and participating in EoL decisions [12,34]. Yet educators’ ability to recognize this distress—and respond appropriately—is shaped not only by how they perceive student engagement but also by their own emotional capacity and institutional constraints. These factors may limit their capacity to offer equitable, individualized support, especially when students’ needs are not overtly expressed.

Our previous research suggests that students’ lack of engagement with EoL care might not indicate a lack of interest but rather an overwhelmed response to these demanding situations [12]. Unfortunately, some medical educators interpret disengagement as a lack of interest, thereby further decreasing support for those students who might need it the most. This misalignment between students’ actual experiences and educators’ perceptions may not only inadvertently hinder the development of empathetic, patient-centered care skills among students [35,36], but also raise important questions about equity in medical education. When educators assess engagement through narrow markers such as confidence, verbal enthusiasm, or emotional expressiveness, there is a risk of conflating personality traits with ethical readiness or clinical potential. Students who are introverted, uncertain, or emotionally restrained — especially when grappling with morally complex scenarios like EoL care — may be misperceived as ethically unfit or less capable. These subtle misjudgments can lead to unequal educational opportunities and inadvertently reinforce structural inequities within clinical training [37,38]. As medical education moves toward more inclusive educational models, it becomes essential to interrogate the implicit norms underlying educator expectations and to invest in faculty development initiatives that foster reflexivity, inclusivity, and epistemic humility in educator–student relationships [39,40,41].

These equity concerns expose a deeper misalignment between how educators perceive student engagement and what students are actually experiencing. However, to understand this gap, we must also acknowledge at least two important factors that compound the complexity of supervising students in emotionally charged situations like EoL care. First, medical educators, who guide students through the complexities of EoL care, face their own set of challenges [42,43]. These include cognitive load and emotional labor from EoL decision-making [44,45], alongside time constraints [46,47], and administrative responsibilities [47,48]. These pressures, while often invisible, may hinder medical educators’ ability to recognize students’ more subtle expressions of distress and provide consistent, individualized support [47,49]. Second, there’s a significant lack of discussion about the disconnect between what students are feeling and what educators perceive. As a result, many educators remain unaware of the internal struggles students experience — including emotional overwhelm, moral dilemmas, and decision-making cognitive load [40].

Notably, both students and medical educators seem to face similar challenges—a theme that clearly emerged in our previous work with students and appears to resonate with some patterns found in this study [12,22,50]. Both students and educators juggle professional responsibilities while dealing with high cognitive load and emotional labor. Amid these pressures, medical educators must navigate a complex tension between the support they aspire to provide and the constraints imposed by their work environment [51,52]. Many described feelings of guilt, frustration, and inadequacy when unable to offer the kind of individualized support they value—especially in the face of time pressure, competing clinical and academic duties, and a hidden curriculum that privileges technical performance. These internal conflicts, though often overlooked, shape the quality and consistency of support they are able to provide [52].

Fostering democratic and dialogical conversation around these shared struggles could create a sense of shared vulnerability, which could offer a unique opportunity for bonding, meaning-making, and a renewed sense of purpose. Paulo Freire, the Brazilian educator, stressed the importance of educators recognizing their unfinishedness as a way to be open to others and feed their curiosity [53]. If this openness is enriched with self-criticism, teachers may create the conditions to be transformed by their interactions with students. According to Freire, when students recognize their impact on such open teachers, they respond by opening themselves to go through the same transformative journey [53,54]. This shared transformative journey may bring a sense of joy that may inspire and refresh students and educators alike [55]. In the next session, we will reflect on how to establish these relationships.

### Practical Implications

*Cultivating the Educational Alliance* – Creating environments that foster democratic dialogue between educators and students is crucial [55]. These efforts shape an educational landscape where honest conversations about the cognitive load and the emotional labor of EoL care could take center stage [56]. Initiatives like monthly reflective sessions stand out by possibly providing a platform for educators and students to jointly navigate knowledge co-construction and mutual learning [57]. These sessions should go beyond mere dialogue; they could be instrumental in building trusting relationships [58]. Such relationships are the cornerstone of the ‘Educational Alliance’—a concept that hinges on a secure and supportive connection between educator and student [58]. In practical terms, strengthening the educational alliance may help realign mismatched perceptions between students and educators, enabling more tailored support and promoting professional growth [57]. Exploring the educational alliance reveals its transformative potential: turning educational encounters into opportunities for compassionate understanding and collective navigation of challenging topics in EoL care [58,59].

*Harnessing Vulnerability to Strengthen Educational Bonds* – Deepening connections between students and medical educators requires a culture that embraces the sharing of personal vulnerability stories in EoL care situations [60]. Imagine a medical educator deliberating over the use of a nasoenteral tube for a comatose patient, weighing its potential benefits against the possibility of merely prolonging suffering. By openly sharing their uncertainties and the emotional labor of balancing personal, family, and team emotions, medical educators showcase vulnerability as a potent tool for connection. Brené Brown’s research underscores vulnerability as a cornerstone for building trust and fostering deep connections, highlighting its power to transform educational relationships [61,62]. Such open dialogues merge personal and professional worlds, crafting bonds that are not only stronger but also more meaningful.

*Empowering Educators through Reflective Practice* – Incorporating reflective practice into faculty development may significantly enhance the culture of connection and growth among educators [63]. Drawing on Steinert’s advocacy for critical self-evaluation, the integration of reflective practices allows medical educators to better align their expectations with the practical realities of their roles [64,65]. By participating in workshops designed to develop adaptive teaching strategies attuned to the challenges of EoL care, educators are encouraged to tailor their teaching methods to meet student needs more effectively. This creates a learning environment that is adaptive, responsive, and supportive [63,64]. Rather than prescribing universal teaching models, reflective practice encourages educators to remain attentive to students’ emotional signals and to navigate uncertainty with openness and flexibility. Together, these strategies strive to bridge the gap between idealized educational visions and the lived realities of the clinical learning environment.

*Structural Commitments for Sustainable Education –* While reflective practice equips individual educators to adapt and grow, it must be supported by institutional structures. Relying on medical educators to carry the cognitive, moral, and emotional weight of end-of-life decisions—while also supporting students on a personal level — depends on institutional commitment. Medical educators need time, space, and structured opportunities to reflect, reconnect, and recharge. This includes setting aside protected time for educational reflection, creating regular opportunities for peer dialogue [66], and creating space within faculty development programs for discussions about emotional labor and moral complexity [67]. When these practices are embedded systemically, they not only strengthen teaching but also signal that the emotional demands of education are institutionally recognized. Evidence from workplace-based peer reflection groups and reflective supervision shows that such practices can reduce emotional exhaustion and improve educators’ sense of efficacy and connection [51]. Such structural support is foundational—not optional—to sustain humanistic, learner-centered environments in health professions education.

### Limitations

Our study has limitations that warrant consideration. First, the cross-sectional design provides a snapshot of educators’ and students’ experiences, restricting our ability to track changes over time or infer causal relationships. Second, the research confines itself to a single Brazilian medical school, immersed in a unique socio-cultural and educational context. This specific setting profoundly influences both pedagogical strategies and medical practice, mainly through the early and intensive clinical engagement characteristic of medical education in Brazil. Such a model, alongside the integrated approach to end-of-life care within general hospital wards—attributable to the absence of dedicated palliative care units—may limit the applicability of our findings across diverse healthcare and educational contexts internationally. Finally, despite meticulous translation efforts by a linguistic specialist, the potential loss of subtle cultural meanings and context-specific nuances during the translation of interviews from Portuguese to English could impact the depth of understanding regarding educators’ support strategies.

## Conclusion

Our findings underscore a crucial insight: misinterpreting students who are emotionally and cognitively overwhelmed as disengaged may inadvertently hamper educators’ support strategies. In response, we propose cultivating an ‘Educational Alliance’ rooted in trust and mutual respect. This alliance beckons educators to embrace vulnerability, deepening connections with students. Yet such openness requires institutional care. When educators are expected to carry the emotional and pedagogical weight of complex teaching scenarios without adequate support, the very foundation of learner-centered education is threatened. Recognizing and addressing the emotional demands placed on educators is not an adjunct to medical education reform—it is central to it. It is time to champion an educational shift—towards a more insightful, responsive, and compassionate approach in medical education, particularly in the delicate realm of EoL care. At the heart of vulnerability lies the transformative power of education.

## Additional File

The additional file for this article can be found as follows:

10.5334/pme.1623.s1 Supplementary Material. Interview Guide.
