# Supplementary material for: Navigating the Tensions of Supervision: Medical Educators’ Strategies for Supporting Students During End-of-Life Care
Source: Perspect Med Educ. 2025 Nov 5;14(1):710–24. doi: 10.5334/pme.1623 (PMC12594084; doi:10.5334/pme.1623)
Supplement: Supplementary Material. — Interview Guide. [file pme-14-1-1623-s1.pdf]

Supplementary Material – Interview Guide
Original & Evolved During Study

Introduction

In end-of-life care, healthcare professionals often face difficult decisions when it comes to life-sustaining treatments. These decisions can be emotionally intense and morally challenging. Two key terms used in such situations are 'withholding' and 'withdrawing' life-sustaining therapies. 'Withholding' treatment means not escalating therapies, such as maintaining current treatment levels even as a terminally ill patient's condition worsens. 'Withdrawing' therapy, on the other hand, refers to discontinuing a treatment initially meant to sustain life, such as suspending mechanical ventilation. These decisions are not only complex but also profoundly impactful on healthcare professionals, including medical students who are in the process of developing their professional identities.

Moral dilemmas are questions that we encounter frequently, where we have to make a choice between issues that affect our freedom of choice and the freedom of others. Our choices often pivot between the necessity of adhering to established norms and the aspiration to uphold personal values and ideals of conduct, which we not only impose upon ourselves but also expect others to acknowledge and follow or, at the very least, accept. This leads us to navigate situations that reveal deep uncertainties and doubts about the best course of action, where none of the available options may seem entirely satisfactory, yet we must still choose the best course.

In this study, we aim to explore how you (medical educators) deal with medical students who are navigating moral dilemmas during End-of-Life care.

Original Interview Guide

Regarding students' reactions during clinical experiences about decision-making processes to withhold or withdraw life-sustaining therapies, I would like to ask:

1. How do you think students handle these situations?
2. Could you share an example from your experience as an educator to illustrate how students handled such a situation?
3. How do you believe students cope with these situations as they navigate through them? What factors do you think influence their coping mechanisms?
4. How do you perceive the dynamics of the student's interactions during these situations? Is it a harmonious experience? For example, how do students interact and relate to the various participants involved, such as supervisors, patients, patients' families, peers, and members of the multidisciplinary team?
5. In your opinion, how do you think students feel while experiencing these situations?
6. Why do you think they feel that way?
7. How do students seem to manage their feelings in these situations?
8. What do you usually do when you notice a student feeling that way?
9. What do you think students take away from these experiences in the short and long term?
10. How do you think these experiences shape the students' development as future professionals?

Interview Guide – Evolution During Iterative Data Collection and Analysis

Regarding students' reactions during clinical experiences about decision-making processes to withhold or withdraw life-sustaining therapies, I would like to ask:

1. How do you think students handle these situations? **[Retained]**

2. Could you share an example from your experience as an educator to illustrate how students handled such a situation? **[Retained]**
3. How do you believe students cope with these situations as they navigate through them? What factors do you think influence their coping mechanisms? **[Deleted during analysis]**
4. How do you perceive the dynamics of the student's interactions during these situations? Is it a harmonious experience? For example, how do students interact and relate to the various participants involved, such as supervisors, patients, patients' families, peers, and members of the multidisciplinary team? **[Deleted during analysis]**
5. In your opinion, how do you think students feel while experiencing these situations? **[Deleted during analysis]**
6. Why do you think they feel that way? **[Deleted during analysis]**
7. How do students seem to manage their feelings in these situations? **[Retained]**
8. What do you usually do when you notice a student feeling that way? **[Retained]**
9. What do you think students take away from these experiences in the short and long term? **[Retained]**
10. How do you think these experiences shape the students' development as future professionals? **[Retained]**
11. Can you describe a specific strategy or approach you use to support students in morally complex end-of-life situations? **[Added later]**
12. In your experience, do students tend to react differently to these situations? What do you do? **[Added later]**

13. How do time constraints or clinical pressures affect the way you support students during these situations? **[Added later]**
14. Have you ever experienced a moment when you felt unable to offer the support a student needed? What happened? **[Added later]**
